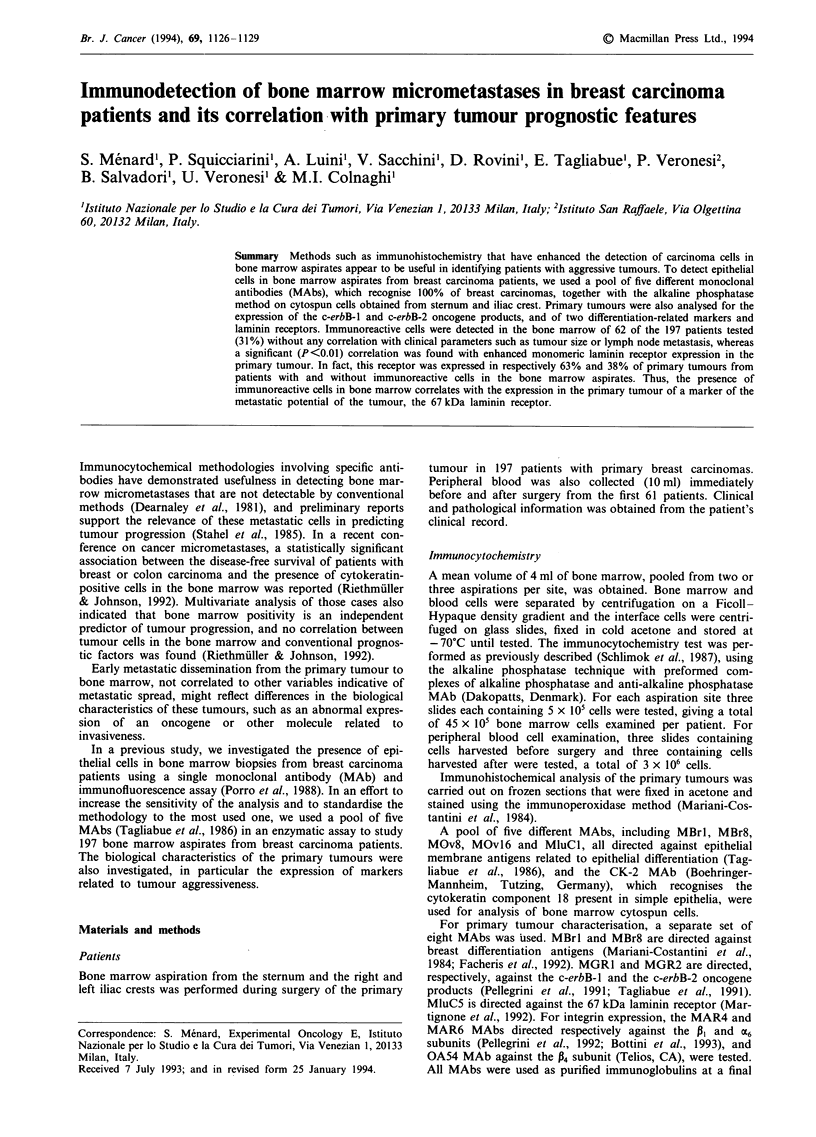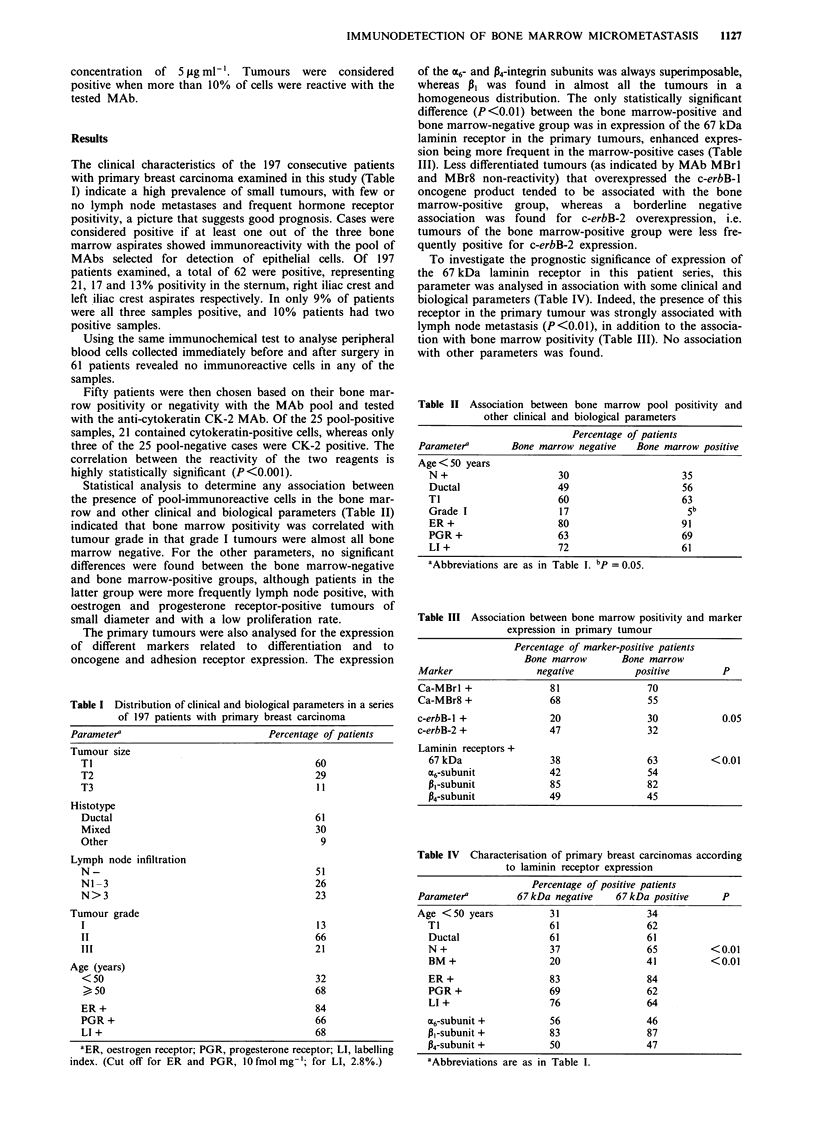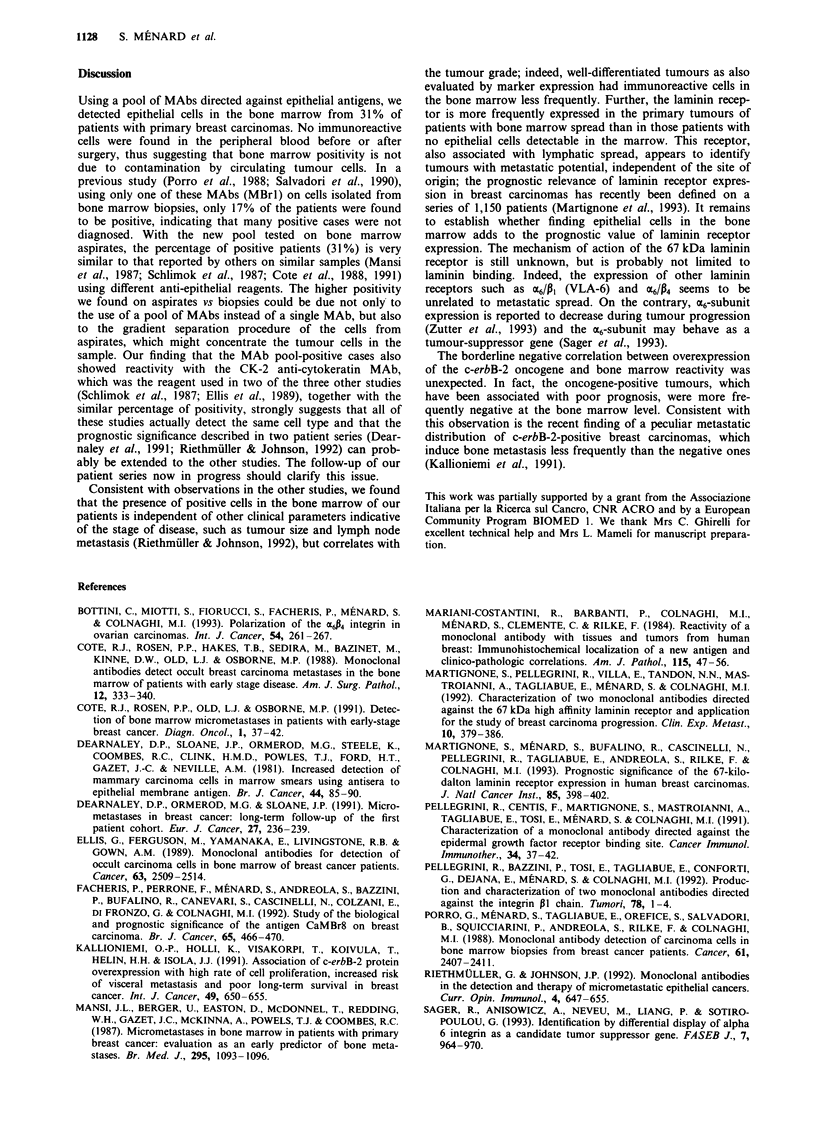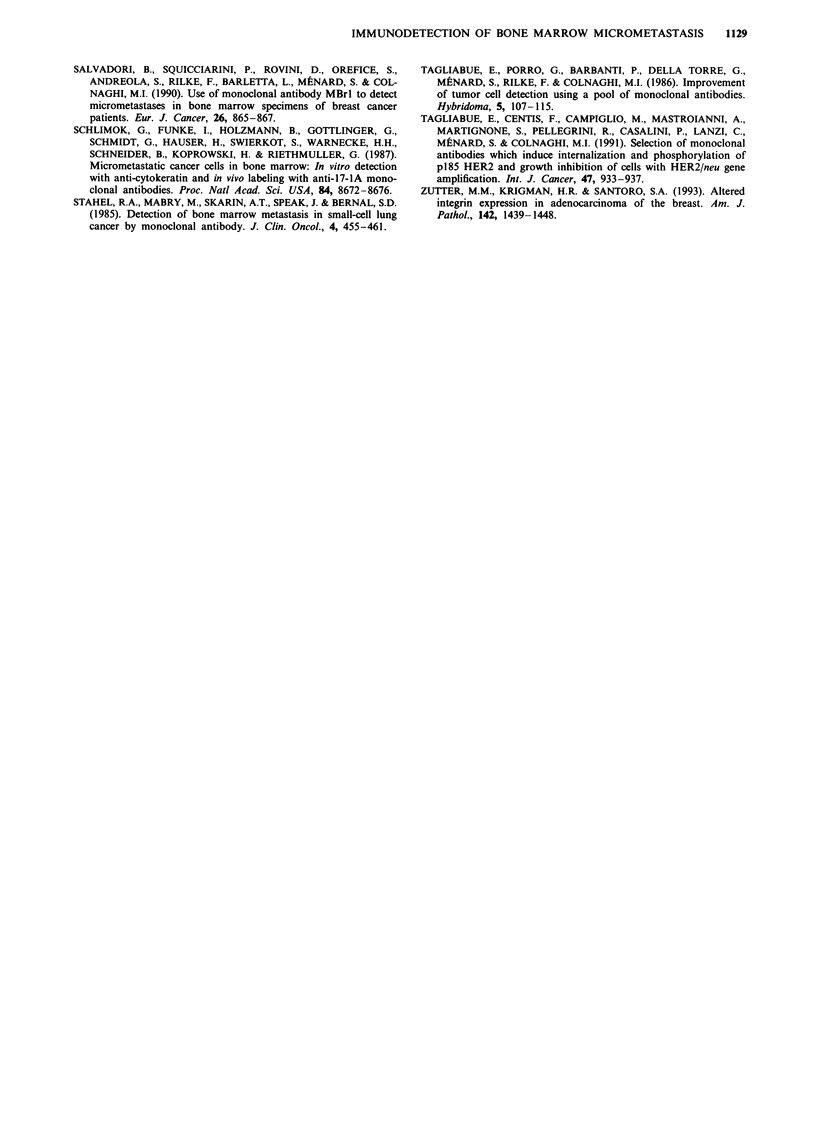# Immunodetection of bone marrow micrometastases in breast carcinoma patients and its correlation with primary tumour prognostic features.

**DOI:** 10.1038/bjc.1994.221

**Published:** 1994-06

**Authors:** S. Ménard, P. Squicciarini, A. Luini, V. Sacchini, D. Rovini, E. Tagliabue, P. Veronesi, B. Salvadori, U. Veronesi, M. I. Colnaghi

**Affiliations:** Istituto Nazionale per lo Studio e la Cura dei Tumori, Milan, Italy.

## Abstract

Methods such as immunohistochemistry that have enhanced the detection of carcinoma cells in bone marrow aspirates appear to be useful in identifying patients with aggressive tumours. To detect epithelial cells in bone marrow aspirates from breast carcinoma patients, we used a pool of five different monoclonal antibodies (MAbs), which recognise 100% of breast carcinomas, together with the alkaline phosphatase method on cytospun cells obtained from sternum and iliac crest. Primary tumours were also analysed for the expression of the c-erbB-1 and c-erbB-2 oncogene products, and of two differentiation-related markers and laminin receptors. Immunoreactive cells were detected in the bone marrow of 62 of the 197 patients tested (31%) without any correlation with clinical parameters such as tumour size or lymph node metastasis, whereas a significant (P < 0.01) correlation was found with enhanced monomeric laminin receptor expression in the primary tumour. In fact, this receptor was expressed in respectively 63% and 38% of primary tumours from patients with and without immunoreactive cells in the bone marrow aspirates. Thus, the presence of immunoreactive cells in bone marrow correlates with the expression in the primary tumour of a marker of the metastatic potential of the tumour, the 67 kDa laminin receptor.


Br. J. Cancer (1994), 69, 1126–1129 © Macmillan Press Ltd., 1994

### Immunodetection of bone marrow micrometastases in breast carcinoma patients and its correlation with primary tumour prognostic features

S. Ménard[1], P. Squicciarini[1], A. Luini[1], V. Sacchini[1], D. Rovini[1], E. Tagliabue[1], P. Veronesi[2], B. Salvadori[1], U. Veronesi[1] & M.I. Colnaghi[1]

[1]*Istituto Nazionale per lo Studio e la Cura dei Tumori, Via Venezian 1, 20133 Milan, Italy;* [2]*Istituto San Raffaele, Via Olgettina 60, 20132 Milan, Italy.*

**Summary** Methods such as immunohistochemistry that have enhanced the detection of carcinoma cells in bone marrow aspirates appear to be useful in identifying patients with aggressive tumours. To detect epithelial cells in bone marrow aspirates from breast carcinoma patients, we used a pool of five different monoclonal antibodies (MAbs), which recognise 100% of breast carcinomas, together with the alkaline phosphatase method on cytospun cells obtained from sternum and iliac crest. Primary tumours were also analysed for the expression of the c-*erb*B-1 and c-*erb*B-2 oncogene products, and of two differentiation-related markers and laminin receptors. Immunoreactive cells were detected in the bone marrow of 62 of the 197 patients tested (31%) without any correlation with clinical parameters such as tumour size or lymph node metastasis, whereas a significant ($P<0.01$) correlation was found with enhanced monomeric laminin receptor expression in the primary tumour. In fact, this receptor was expressed in respectively 63% and 38% of primary tumours from patients with and without immunoreactive cells in the bone marrow aspirates. Thus, the presence of immunoreactive cells in bone marrow correlates with the expression in the primary tumour of a marker of the metastatic potential of the tumour, the 67 kDa laminin receptor.

Immunocytochemical methodologies involving specific antibodies have demonstrated usefulness in detecting bone marrow micrometastases that are not detectable by conventional methods (Dearnaley *et al.*, 1981), and preliminary reports support the relevance of these metastatic cells in predicting tumour progression (Stahel *et al.*, 1985). In a recent conference on cancer micrometastases, a statistically significant association between the disease-free survival of patients with breast or colon carcinoma and the presence of cytokeratin-positive cells in the bone marrow was reported (Riethmüller & Johnson, 1992). Multivariate analysis of those cases also indicated that bone marrow positivity is an independent predictor of tumour progression, and no correlation between tumour cells in the bone marrow and conventional prognostic factors was found (Riethmüller & Johnson, 1992).

Early metastatic dissemination from the primary tumour to bone marrow, not correlated to other variables indicative of metastatic spread, might reflect differences in the biological characteristics of these tumours, such as an abnormal expression of an oncogene or other molecule related to invasiveness.

In a previous study, we investigated the presence of epithelial cells in bone marrow biopsies from breast carcinoma patients using a single monoclonal antibody (MAb) and immunofluorescence assay (Porro *et al.*, 1988). In an effort to increase the sensitivity of the analysis and to standardise the methodology to the most used one, we used a pool of five MAbs (Tagliabue *et al.*, 1986) in an enzymatic assay to study 197 bone marrow aspirates from breast carcinoma patients. The biological characteristics of the primary tumours were also investigated, in particular the expression of markers related to tumour aggressiveness.

### Materials and methods

### *Patients*

Bone marrow aspiration from the sternum and the right and left iliac crests was performed during surgery of the primary tumour in 197 patients with primary breast carcinomas. Peripheral blood was also collected (10 ml) immediately before and after surgery from the first 61 patients. Clinical and pathological information was obtained from the patient's clinical record.

### *Immunocytochemistry*

A mean volume of 4 ml of bone marrow, pooled from two or three aspirations per site, was obtained. Bone marrow and blood cells were separated by centrifugation on a Ficoll–Hypaque density gradient and the interface cells were centrifuged on glass slides, fixed in cold acetone and stored at $-70$°C until tested. The immunocytochemistry test was performed as previously described (Schlimok *et al.*, 1987), using the alkaline phosphatase technique with preformed complexes of alkaline phosphatase and anti-alkaline phosphatase MAb (Dakopatts, Denmark). For each aspiration site three slides each containing $5 \times 10^5$ cells were tested, giving a total of $45 \times 10^5$ bone marrow cells examined per patient. For peripheral blood cell examination, three slides containing cells harvested before surgery and three containing cells harvested after were tested, a total of $3 \times 10^6$ cells.

Immunohistochemical analysis of the primary tumours was carried out on frozen sections that were fixed in acetone and stained using the immunoperoxidase method (Mariani-Costantini *et al.*, 1984).

A pool of five different MAbs, including MBr1, MBr8, MOv8, MOv16 and MluC1, all directed against epithelial membrane antigens related to epithelial differentiation (Tagliabue *et al.*, 1986), and the CK-2 MAb (Boehringer-Mannheim, Tutzing, Germany), which recognises the cytokeratin component 18 present in simple epithelia, were used for analysis of bone marrow cytospun cells.

For primary tumour characterisation, a separate set of eight MAbs was used. MBr1 and MBr8 are directed against breast differentiation antigens (Mariani-Costantini *et al.*, 1984; Facheris *et al.*, 1992). MGR1 and MGR2 are directed, respectively, against the c-*erb*B-1 and the c-*erb*B-2 oncogene products (Pellegrini *et al.*, 1991; Tagliabue *et al.*, 1991). MluC5 is directed against the 67 kDa laminin receptor (Martignone *et al.*, 1992). For integrin expression, the MAR4 and MAR6 MAbs directed respectively against the $\beta_1$ and $\alpha_6$ subunits (Pellegrini *et al.*, 1992; Bottini *et al.*, 1993), and OA54 MAb against the $\beta_4$ subunit (Telios, CA), were tested. All MAbs were used as purified immunoglobulins at a final

Correspondence: S. Ménard, Experimental Oncology E, Istituto Nazionale per lo Studio e la Cura dei Tumori, Via Venezian 1, 20133 Milan, Italy.
Received 7 July 1993; and in revised form 25 January 1994.

concentration of $5\,\mu g\,ml^{-1}$. Tumours were considered positive when more than 10% of cells were reactive with the tested MAb.

### Results

The clinical characteristics of the 197 consecutive patients with primary breast carcinoma examined in this study (Table I) indicate a high prevalence of small tumours, with few or no lymph node metastases and frequent hormone receptor positivity, a picture that suggests good prognosis. Cases were considered positive if at least one out of the three bone marrow aspirates showed immunoreactivity with the pool of MAbs selected for detection of epithelial cells. Of 197 patients examined, a total of 62 were positive, representing 21, 17 and 13% positivity in the sternum, right iliac crest and left iliac crest aspirates respectively. In only 9% of patients were all three samples positive, and 10% patients had two positive samples.

Using the same immunochemical test to analyse peripheral blood cells collected immediately before and after surgery in 61 patients revealed no immunoreactive cells in any of the samples.

Fifty patients were then chosen based on their bone marrow positivity or negativity with the MAb pool and tested with the anti-cytokeratin CK-2 MAb. Of the 25 pool-positive samples, 21 contained cytokeratin-positive cells, whereas only three of the 25 pool-negative cases were CK-2 positive. The correlation between the reactivity of the two reagents is highly statistically significant ($P<0.001$).

Statistical analysis to determine any association between the presence of pool-immunoreactive cells in the bone marrow and other clinical and biological parameters (Table II) indicated that bone marrow positivity was correlated with tumour grade in that grade I tumours were almost all bone marrow negative. For the other parameters, no significant differences were found between the bone marrow-negative and bone marrow-positive groups, although patients in the latter group were more frequently lymph node positive, with oestrogen and progesterone receptor-positive tumours of small diameter and with a low proliferation rate.

The primary tumours were also analysed for the expression of different markers related to differentiation and to oncogene and adhesion receptor expression. The expression of the $\alpha_6$- and $\beta_4$-integrin subunits was always superimposable, whereas $\beta_1$ was found in almost all the tumours in a homogeneous distribution. The only statistically significant difference ($P<0.01$) between the bone marrow-positive and bone marrow-negative group was in expression of the 67 kDa laminin receptor in the primary tumours, enhanced expression being more frequent in the marrow-positive cases (Table III). Less differentiated tumours (as indicated by MAb MBr1 and MBr8 non-reactivity) that overexpressed the c-*erb*B-1 oncogene product tended to be associated with the bone marrow-positive group, whereas a borderline negative association was found for c-*erb*B-2 overexpression, i.e. tumours of the bone marrow-positive group were less frequently positive for c-*erb*B-2 expression.

To investigate the prognostic significance of expression of the 67 kDa laminin receptor in this patient series, this parameter was analysed in association with some clinical and biological parameters (Table IV). Indeed, the presence of this receptor in the primary tumour was strongly associated with lymph node metastasis ($P<0.01$), in addition to the association with bone marrow positivity (Table III). No association with other parameters was found.

**Table I** Distribution of clinical and biological parameters in a series of 197 patients with primary breast carcinoma

| *Parameter*[a] | *Percentage of patients* |
|---|---|
| Tumour size | |
| T1 | 60 |
| T2 | 29 |
| T3 | 11 |
| Histotype | |
| Ductal | 61 |
| Mixed | 30 |
| Other | 9 |
| Lymph node infiltration | |
| N − | 51 |
| N1-3 | 26 |
| N>3 | 23 |
| Tumour grade | |
| I | 13 |
| II | 66 |
| III | 21 |
| Age (years) | |
| <50 | 32 |
| ⩾50 | 68 |
| ER + | 84 |
| PGR + | 66 |
| LI + | 68 |

[a]ER, oestrogen receptor; PGR, progesterone receptor; LI, labelling index. (Cut off for ER and PGR, $10\,fmol\,mg^{-1}$; for LI, 2.8%.)

**Table II** Association between bone marrow pool positivity and other clinical and biological parameters

| | *Percentage of patients* | |
|---|---|---|
| *Parameter*[a] | *Bone marrow negative* | *Bone marrow positive* |
| Age<50 years | | |
| N + | 30 | 35 |
| Ductal | 49 | 56 |
| T1 | 60 | 63 |
| Grade I | 17 | 5[b] |
| ER + | 80 | 91 |
| PGR + | 63 | 69 |
| LI + | 72 | 61 |

[a]Abbreviations are as in Table I. [b]$P = 0.05$.

**Table III** Association between bone marrow positivity and marker expression in primary tumour

| | *Percentage of marker-positive patients* | | |
|---|---|---|---|
| *Marker* | *Bone marrow negative* | *Bone marrow positive* | *P* |
| Ca-MBr1 + | 81 | 70 | |
| Ca-MBr8 + | 68 | 55 | |
| c-*erb*B-1 + | 20 | 30 | 0.05 |
| c-*erb*B-2 + | 47 | 32 | |
| Laminin receptors + | | | |
| 67 kDa | 38 | 63 | <0.01 |
| $\alpha_6$-subunit | 42 | 54 | |
| $\beta_1$-subunit | 85 | 82 | |
| $\beta_4$-subunit | 49 | 45 | |

**Table IV** Characterisation of primary breast carcinomas according to laminin receptor expression

| | *Percentage of positive patients* | | |
|---|---|---|---|
| *Parameter*[a] | *67 kDa negative* | *67 kDa positive* | *P* |
| Age <50 years | 31 | 34 | |
| T1 | 61 | 62 | |
| Ductal | 61 | 61 | |
| N + | 37 | 65 | <0.01 |
| BM + | 20 | 41 | <0.01 |
| ER + | 83 | 84 | |
| PGR + | 69 | 62 | |
| LI + | 76 | 64 | |
| $\alpha_6$-subunit + | 56 | 46 | |
| $\beta_1$-subunit + | 83 | 87 | |
| $\beta_4$-subunit + | 50 | 47 | |

[a]Abbreviations are as in Table I.

### Discussion

Using a pool of MAbs directed against epithelial antigens, we detected epithelial cells in the bone marrow from 31% of patients with primary breast carcinomas. No immunoreactive cells were found in the peripheral blood before or after surgery, thus suggesting that bone marrow positivity is not due to contamination by circulating tumour cells. In a previous study (Porro *et al.*, 1988; Salvadori *et al.*, 1990), using only one of these MAbs (MBr1) on cells isolated from bone marrow biopsies, only 17% of the patients were found to be positive, indicating that many positive cases were not diagnosed. With the new pool tested on bone marrow aspirates, the percentage of positive patients (31%) is very similar to that reported by others on similar samples (Mansi *et al.*, 1987; Schlimok *et al.*, 1987; Cote *et al.*, 1988, 1991) using different anti-epithelial reagents. The higher positivity we found on aspirates *vs* biopsies could be due not only to the use of a pool of MAbs instead of a single MAb, but also to the gradient separation procedure of the cells from aspirates, which might concentrate the tumour cells in the sample. Our finding that the MAb pool-positive cases also showed reactivity with the CK-2 anti-cytokeratin MAb, which was the reagent used in two of the three other studies (Schlimok *et al.*, 1987; Ellis *et al.*, 1989), together with the similar percentage of positivity, strongly suggests that all of these studies actually detect the same cell type and that the prognostic significance described in two patient series (Dearnaley *et al.*, 1991; Riethmüller & Johnson, 1992) can probably be extended to the other studies. The follow-up of our patient series now in progress should clarify this issue.

Consistent with observations in the other studies, we found that the presence of positive cells in the bone marrow of our patients is independent of other clinical parameters indicative of the stage of disease, such as tumour size and lymph node metastasis (Riethmüller & Johnson, 1992), but correlates with the tumour grade; indeed, well-differentiated tumours as also evaluated by marker expression had immunoreactive cells in the bone marrow less frequently. Further, the laminin receptor is more frequently expressed in the primary tumours of patients with bone marrow spread than in those patients with no epithelial cells detectable in the marrow. This receptor, also associated with lymphatic spread, appears to identify tumours with metastatic potential, independent of the site of origin; the prognostic relevance of laminin receptor expression in breast carcinomas has recently been defined on a series of 1,150 patients (Martignone *et al.*, 1993). It remains to establish whether finding epithelial cells in the bone marrow adds to the prognostic value of laminin receptor expression. The mechanism of action of the 67 kDa laminin receptor is still unknown, but is probably not limited to laminin binding. Indeed, the expression of other laminin receptors such as $\alpha_6/\beta_1$ (VLA-6) and $\alpha_6/\beta_4$ seems to be unrelated to metastatic spread. On the contrary, $\alpha_6$-subunit expression is reported to decrease during tumour progression (Zutter *et al.*, 1993) and the $\alpha_6$-subunit may behave as a tumour-suppressor gene (Sager *et al.*, 1993).

The borderline negative correlation between overexpression of the c-*erb*B-2 oncogene and bone marrow reactivity was unexpected. In fact, the oncogene-positive tumours, which have been associated with poor prognosis, were more frequently negative at the bone marrow level. Consistent with this observation is the recent finding of a peculiar metastatic distribution of c-*erb*B-2-positive breast carcinomas, which induce bone metastasis less frequently than the negative ones (Kallioniemi *et al.*, 1991).

This work was partially supported by a grant from the Associazione Italiana per la Ricerca sul Cancro, CNR ACRO and by a European Community Program BIOMED 1. We thank Mrs C. Ghirelli for excellent technical help and Mrs L. Mameli for manuscript preparation.

### References

BOTTINI, C., MIOTTI, S., FIORUCCI, S., FACHERIS, P., MÉNARD, S. & COLNAGHI, M.I. (1993). Polarization of the $\alpha_6\beta_4$ integrin in ovarian carcinomas. *Int. J. Cancer*, **54**, 261–267.

COTE, R.J., ROSEN, P.P., HAKES, T.B., SEDIRA, M., BAZINET, M., KINNE, D.W., OLD, L.J. & OSBORNE, M.P. (1988). Monoclonal antibodies detect occult breast carcinoma metastases in the bone marrow of patients with early stage disease. *Am. J. Surg. Pathol.*, **12**, 333–340.

COTE, R.J., ROSEN, P.P., OLD, L.J. & OSBORNE, M.P. (1991). Detection of bone marrow micrometastases in patients with early-stage breast cancer. *Diagn. Oncol.*, **1**, 37–42.

DEARNALEY, D.P., SLOANE, J.P., ORMEROD, M.G., STEELE, K., COOMBES, R.C., CLINK, H.M.D., POWLES, T.J., FORD, H.T., GAZET, J.-C. & NEVILLE, A.M. (1981). Increased detection of mammary carcinoma cells in marrow smears using antisera to epithelial membrane antigen. *Br. J. Cancer*, **44**, 85–90.

DEARNALEY, D.P., ORMEROD, M.G. & SLOANE, J.P. (1991). Micrometastases in breast cancer: long-term follow-up of the first patient cohort. *Eur. J. Cancer*, **27**, 236–239.

ELLIS, G., FERGUSON, M., YAMANAKA, E., LIVINGSTONE, R.B. & GOWN, A.M. (1989). Monoclonal antibodies for detection of occult carcinoma cells in bone marrow of breast cancer patients. *Cancer*, **63**, 2509–2514.

FACHERIS, P., PERRONE, F., MÉNARD, S., ANDREOLA, S., BAZZINI, P., BUFALINO, R., CANEVARI, S., CASCINELLI, N., COLZANI, E., DI FRONZO, G. & COLNAGHI, M.I. (1992). Study of the biological and prognostic significance of the antigen CaMBr8 on breast carcinoma. *Br. J. Cancer*, **65**, 466–470.

KALLIONIEMI, O.-P., HOLLI, K., VISAKORPI, T., KOIVULA, T., HELIN, H.H. & ISOLA, J.J. (1991). Association of c-*erb*B-2 protein overexpression with high rate of cell proliferation, increased risk of visceral metastasis and poor long-term survival in breast cancer. *Int. J. Cancer*, **49**, 650–655.

MANSI, J.L., BERGER, U., EASTON, D., MCDONNEL, T., REDDING, W.H., GAZET, J.C., MCKINNA, A., POWELS, T.J. & COOMBES, R.C. (1987). Micrometastases in bone marrow in patients with primary breast cancer: evaluation as an early predictor of bone metastases. *Br. Med. J.*, **295**, 1093–1096.

MARIANI-COSTANTINI, R., BARBANTI, P., COLNAGHI, M.I., MÉNARD, S., CLEMENTE, C. & RILKE, F. (1984). Reactivity of a monoclonal antibody with tissues and tumors from human breast: Immunohistochemical localization of a new antigen and clinico-pathologic correlations. *Am. J. Pathol.*, **115**, 47–56.

MARTIGNONE, S., PELLEGRINI, R., VILLA, E., TANDON, N.N., MASTROIANNI, A., TAGLIABUE, E., MÉNARD, S. & COLNAGHI, M.I. (1992). Characterization of two monoclonal antibodies directed against the 67 kDa high affinity laminin receptor and application for the study of breast carcinoma progression. *Clin. Exp. Metast.*, **10**, 379–386.

MARTIGNONE, S., MÉNARD, S., BUFALINO, R., CASCINELLI, N., PELLEGRINI, R., TAGLIABUE, E., ANDREOLA, S., RILKE, F. & COLNAGHI, M.I. (1993). Prognostic significance of the 67-kilodalton laminin receptor expression in human breast carcinomas. *J. Natl Cancer Inst.*, **85**, 398–402.

PELLEGRINI, R., CENTIS, F., MARTIGNONE, S., MASTROIANNI, A., TAGLIABUE, E., TOSI, E., MÉNARD, S. & COLNAGHI, M.I. (1991). Characterization of a monoclonal antibody directed against the epidermal growth factor receptor binding site. *Cancer Immunol. Immunother.*, **34**, 37–42.

PELLEGRINI, R., BAZZINI, P., TOSI, E., TAGLIABUE, E., CONFORTI, G., DEJANA, E., MÉNARD, S. & COLNAGHI, M.I. (1992). Production and characterization of two monoclonal antibodies directed against the integrin $\beta 1$ chain. *Tumori*, **78**, 1–4.

PORRO, G., MÉNARD, S., TAGLIABUE, E., OREFICE, S., SALVADORI, B., SQUICCIARINI, P., ANDREOLA, S., RILKE, F. & COLNAGHI, M.I. (1988). Monoclonal antibody detection of carcinoma cells in bone marrow biopsies from breast cancer patients. *Cancer*, **61**, 2407–2411.

RIETHMÜLLER, G. & JOHNSON, J.P. (1992). Monoclonal antibodies in the detection and therapy of micrometastatic epithelial cancers. *Curr. Opin. Immunol.*, **4**, 647–655.

SAGER, R., ANISOWICZ, A., NEVEU, M., LIANG, P. & SOTIROPOULOU, G. (1993). Identification by differential display of alpha 6 integrin as a candidate tumor suppressor gene. *FASEB J.*, **7**, 964–970.

SALVADORI, B., SQUICCIARINI, P., ROVINI, D., OREFICE, S., ANDREOLA, S., RILKE, F., BARLETTA, L., MÉNARD, S. & COLNAGHI, M.I. (1990). Use of monoclonal antibody MBr1 to detect micrometastases in bone marrow specimens of breast cancer patients. *Eur. J. Cancer*, **26**, 865–867.

SCHLIMOK, G., FUNKE, I., HOLZMANN, B., GOTTLINGER, G., SCHMIDT, G., HAUSER, H., SWIERKOT, S., WARNECKE, H.H., SCHNEIDER, B., KOPROWSKI, H. & RIETHMULLER, G. (1987). Micrometastatic cancer cells in bone marrow: *In vitro* detection with anti-cytokeratin and *in vivo* labeling with anti-17-1A monoclonal antibodies. *Proc. Natl Acad. Sci. USA*, **84**, 8672–8676.

STAHEL, R.A., MABRY, M., SKARIN, A.T., SPEAK, J. & BERNAL, S.D. (1985). Detection of bone marrow metastasis in small-cell lung cancer by monoclonal antibody. *J. Clin. Oncol.*, **4**, 455–461.

TAGLIABUE, E., PORRO, G., BARBANTI, P., DELLA TORRE, G., MÉNARD, S., RILKE, F. & COLNAGHI, M.I. (1986). Improvement of tumor cell detection using a pool of monoclonal antibodies. *Hybridoma*, **5**, 107–115.

TAGLIABUE, E., CENTIS, F., CAMPIGLIO, M., MASTROIANNI, A., MARTIGNONE, S., PELLEGRINI, R., CASALINI, P., LANZI, C., MÉNARD, S. & COLNAGHI, M.I. (1991). Selection of monoclonal antibodies which induce internalization and phosphorylation of p185 HER2 and growth inhibition of cells with HER2/*neu* gene amplification. *Int. J. Cancer*, **47**, 933–937.

ZUTTER, M.M., KRIGMAN, H.R. & SANTORO, S.A. (1993). Altered integrin expression in adenocarcinoma of the breast. *Am. J. Pathol.*, **142**, 1439–1448.